# Supplementary material for: Engineered fluoride sensitivity enables biocontainment and selection of genetically-modified yeasts
Source: Nat Commun. 2020 Oct 29;11:5459. doi: 10.1038/s41467-020-19271-1 (PMC7596524; doi:10.1038/s41467-020-19271-1)
Supplement: Supplementary file 1 — Supplemetnary Information [file 41467_2020_19271_MOESM1_ESM.pdf]

Supplementary Information

Engineered fluoride sensitivity enables biocontainment and selection of genetically-modified yeasts

Yoo et al.

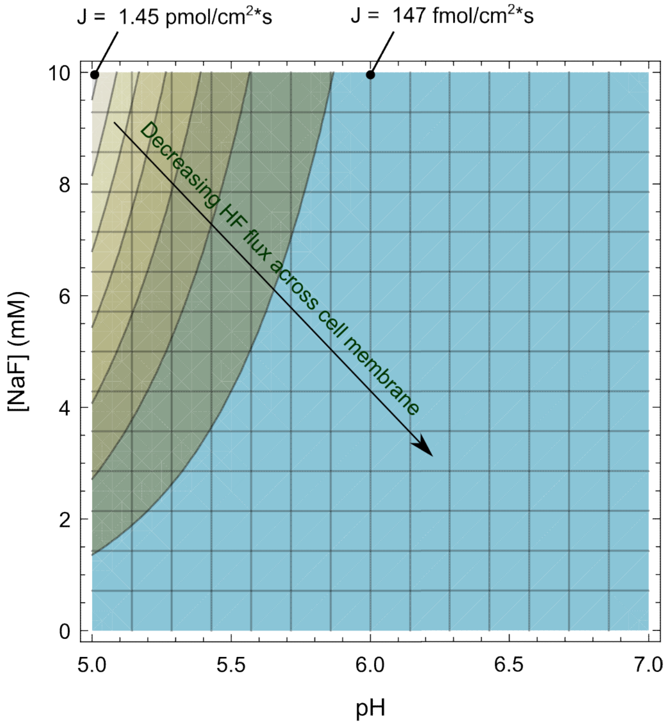

Supplementary Figure 1: A model of fluoride transport across the yeast cell membrane suggests significant influence of pH and NaF concentration on fluoride flux. In the presence of 10 mM NaF, the flux of fluoride across yeast membranes is expected to increase 10-fold upon reducing culture medium by 1 pH unit. In the contour plot, light brown indicated greater fluoride flux and blue indicates lower flux.

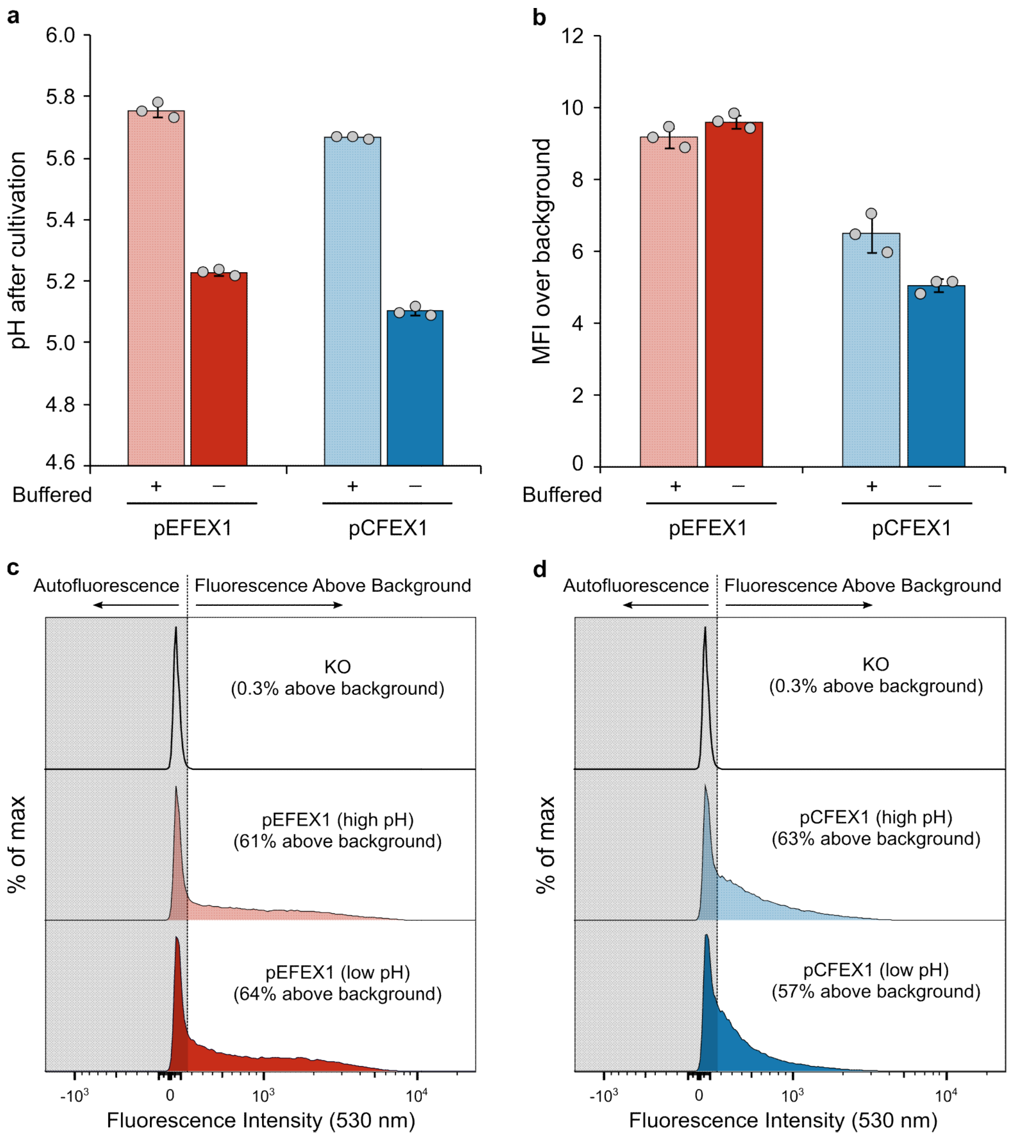

Supplementary Figure 2: Buffering cell medium does not significantly influence cellular phenotype upon expression of *A_2aR-GFP* from pCFEX1 and pEFEX1 vector backbones. (a) Buffering yeast culture media imparts control of pH and, potentially, fluoride flux across the cell membrane. (b - d) However, *A_2aR-GFP* yield and expression phenotypes are similar between buffered and unbuffered cells. Data presented in panels a and b represent the mean of three biological replicates, and error bars represent their standard deviation. Histograms presented in panels c and d correspond to representative samples. Source data are provided as a Source Data file.

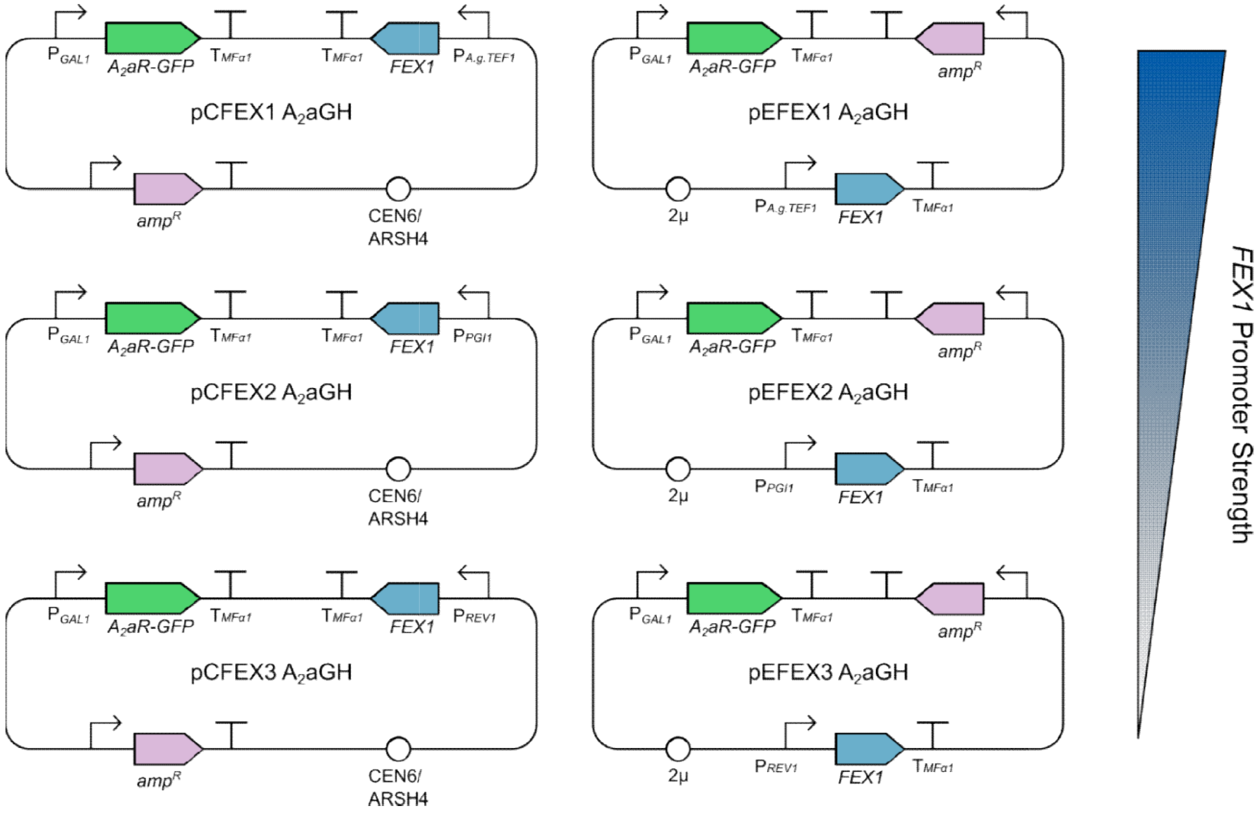

Supplementary Figure 3: Diagrams of pCFEX and pEFEX vectors containing promoters of varying strength driving *FEX1* expression. The non-integrating FEX vectors contain an ampicillin selection marker for cloning in bacteria as well as low- (pCFEX) and high-copy (pEFEX) origins of replication. The vectors are shown containing a P_{GAL1}-driven *A_2aR-GFP* coding sequence as well as a constitutively active promoter driving *FEX1* expression. The constitutive promoters vary in strength ranging from strong ($P_{A.g.\ TEF1}$), to medium (P_{PGI1}), to weak (P_{REV1}).

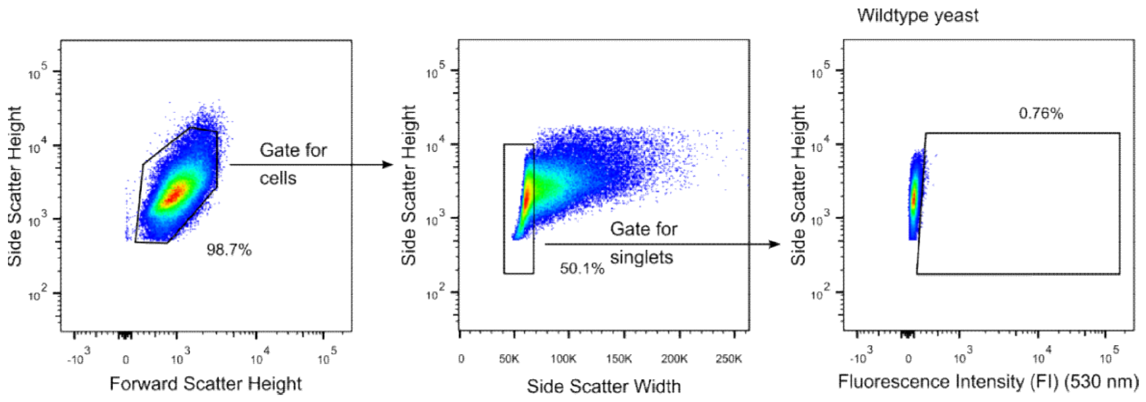

Supplementary Figure 4: Illustration of FACS gating strategy. All yeast samples were gated using the illustrated strategy. Yeast were initially gated to collect cells and singlets. Subsequently, the fluorescence intensity of all singlet cells was measured at 530 nm. The background fluorescence intensity was determined using the wildtype yeast strain or the biocontainment strain containing an empty FEX vector for their corresponding experiments. The proportion of a population exhibiting fluorescence above background was determined through gating the negative control singlet population to capture ~1% of cells with respect to fluorescence intensity measured at 530 nm, as shown in the right-most panel.

Supplementary Table 1: Biocontainment strain escape rates

[NaF]	Escape Rate
210.5 μM	7.81×10^{-9}
5 mM	Below detection limit

Supplementary Table 2: Cost comparison of common yeast selection markers at recommended working concentrations

Compound	Recommended Working Concentration (μg/mL)	Cost (US$/L)
NaF	84 (2 mM)	0.04
G418	300 - 1000	31.95 - 106.50
Canavanine	100	91.20
Hygromycin	200	184.00
Phleomycin	10	151.00
Nourseothricin	100	2175.00

Supplementary Table 3: Primers used in this study

Primer Number	Sequence (5' - 3')
1	CGATTGGAATTCAGATCTGTTTAGCTTGCCTCGT
2	CATTGGCGGCCGGGTTGTTTATGTTCGGATGTG
3	GTACTGCGGCCGATGATTTTCAATCCGGTCATATC
4	CTATCTTAAGCTAACAAATCGGGTTGGTCAATC
5	AGCTGACCUAGATCTGTTTAGCTTGCCTCGT
6	ACTCCTCGUCAATTCTCTTAGGATTCGATTCAC
7	AGGTCAGCUGATGGGTCCTTTTCATCACG
8	ACGAGGAGUAGAGTATGAGTATTCAACATTTCCG
9	AGGTCAGCUCTTATCGATGATAAGCTGTCAAAG
10	AGCTGACCUGTAACAAAAATCACGATCTGGG
11	ATTTTGAATTCU TTTTAGGCTGGTATCTTGATTCTAAA
12	AGCTGACCUGTGTTGTTATCCGATACAACCGG
13	ATTTTGAATTCUCGCTGGATATGCCTAGAAATGC
14	AGAATTCAAAAU GATTTTCAATCCGGTCATATC
15	AGGTCAGCUCGTAGGTATTTGAAGTCACCGG
16	ACGAGGAGUCAATAAGAACATTGCTGATGTGATG
17	AGCAGTACUTTATTATTTGTACAATTCATCCATACCATG
18	AGTACTGCUGTCATGTAATTAGTTATGTCACGC
19	CTCAAGAATCTGCAGTTATTTAATTATTTAATCGAGCGTGTAATGCTC TGTAGGGATAACAGGGTAATCCGCGCGTTGGCCGATTCAT
20	AAAAAAAAAAAGTGGGATGATTATGCAGGGAAAAGTATAAAGAAAA GATCTTCGTACGCTGCAGGTCGAC
21	AAAAAAAAAAGGTGGGATGATTGTGCAGGAAAAGGTATGAAGAAA AGATCTTCGTACGCTGCAGGTCGAC
22	TGCAGTTATTTAATTATTTAATCGAGCGTGTAATGCTCTGGATCTTTT CTTCATACCTTTTCCTGCACAATCATCCCACC
23	GGTGGGATGATTGTGCAGGAAAAGGTATGAAGAAAAGATCCAGAG CATTACACGCTCGATTAAATAATTAAATAACTGCA
