# Supplementary material for: Engineered fluoride sensitivity enables biocontainment and selection of genetically-modified yeasts
Source: Nat Commun. 2020 Oct 29;11:5459. doi: 10.1038/s41467-020-19271-1 (PMC7596524; doi:10.1038/s41467-020-19271-1)
Supplement: Supplementary file 3 — Reporting Summary [file 41467_2020_19271_MOESM3_ESM.pdf]

nature research

Corresponding author(s): Michelle O'Malley

Last updated by author(s): Sep 3, 2020

Reporting Summary

Nature Research wishes to improve the reproducibility of the work that we publish. This form provides structure for consistency and transparency in reporting. For further information on Nature Research policies, see Authors & Referees and the Editorial Policy Checklist .

Statistics

For all statistical analyses, confirm that the following items are present in the figure legend, table legend, main text, or Methods section.

n/a	Confirmed	
☐	☒	The exact sample size (*n*) for each experimental group/condition, given as a discrete number and unit of measurement
☐	☒	A statement on whether measurements were taken from distinct samples or whether the same sample was measured repeatedly
☒	☐	The statistical test(s) used AND whether they are one- or two-sided<br>*Only common tests should be described solely by name; describe more complex techniques in the Methods section.*
☒	☐	A description of all covariates tested
☒	☐	A description of any assumptions or corrections, such as tests of normality and adjustment for multiple comparisons
☐	☒	A full description of the statistical parameters including central tendency (e.g. means) or other basic estimates (e.g. regression coefficient) AND variation (e.g. standard deviation) or associated estimates of uncertainty (e.g. confidence intervals)
☒	☐	For null hypothesis testing, the test statistic (e.g. *F*, *t*, *r*) with confidence intervals, effect sizes, degrees of freedom and *P* value noted<br>*Give P values as exact values whenever suitable.*
☒	☐	For Bayesian analysis, information on the choice of priors and Markov chain Monte Carlo settings
☒	☐	For hierarchical and complex designs, identification of the appropriate level for tests and full reporting of outcomes
☒	☐	Estimates of effect sizes (e.g. Cohen's *d*, Pearson's *r*), indicating how they were calculated

Our web collection on statistics for biologists contains articles on many of the points above.

Software and code

Policy information about availability of computer code

Data collection	No software was used for data collection in this study.
Data analysis	Flow cytometric data was analyzed using FlowJo V.10.0.7r2

For manuscripts utilizing custom algorithms or software that are central to the research but not yet described in published literature, software must be made available to editors/reviewers. We strongly encourage code deposition in a community repository (e.g. GitHub). See the Nature Research guidelines for submitting code & software for further information.

Data

Policy information about availability of data

All manuscripts must include a data availability statement. This statement should provide the following information, where applicable:

- Accession codes, unique identifiers, or web links for publicly available datasets
- A list of figures that have associated raw data
- A description of any restrictions on data availability

The data that support the findings of this study are available from the corresponding author upon reasonable request. The biocontainment strain, BJ5465 fex1::GSHU Δfex2, will be deposited with ATCC and will be available through accession #. The pIFEX, pEFEX, and pCFEX vectors described in this work will be deposited with Addgene. The vectors and associated vector maps will be available through accession #.

Field-specific reporting

Please select the one below that is the best fit for your research. If you are not sure, read the appropriate sections before making your selection.

Life sciences study design

All studies must disclose on these points even when the disclosure is negative.

Sample size	A sample size of 3-4 biological yeast (S. cerevisiae) replicates was used for data presented in the manuscript with the exception of that presented in Figure 3a, which was subject to throughput limitations. Specifically, triplicate or quadruplicate samples were used to collect data represented by Figures 2b-c, e-f, 3e, and Supplemental Figure 2a-b. For flow cytometric analyses, ~60,000 single cells were analyzed for each sample. This sample size is in accordance with literature standards and enables identification of outliers, which were not encountered in our data.
Data exclusions	No data were excluded from this analysis.
Replication	Experiments consisting of 3 independent, biological replicates demonstrated high degrees of reproducibility in each experiment.
Randomization	Randomization was not relevant to this study as samples were not grouped for experiments or analyses. Controls and experimental samples were not sourced from the same group (i.e. strain) thus do not require randomization to account for bias due to grouping.
Blinding	Blinding was not relevant to this study as samples were not grouped for any experiments or analyses thus blinding is not necessary to account for bias due to grouping.

Reporting for specific materials, systems and methods

We require information from authors about some types of materials, experimental systems and methods used in many studies. Here, indicate whether each material, system or method listed is relevant to your study. If you are not sure if a list item applies to your research, read the appropriate section before selecting a response.

Materials & experimental systems

n/a	Involved in the study	
☒	☐	Antibodies
☒	☐	Eukaryotic cell lines
☒	☐	Palaeontology
☒	☐	Animals and other organisms
☒	☐	Human research participants
☒	☐	Clinical data

Methods

n/a	Involved in the study	
☒	☐	ChIP-seq
☒	☐	Flow cytometry
☒	☐	MRI-based neuroimaging
